# Supplementary material for: Modeling glioblastoma heterogeneity as a dynamic network of cell states
Source: Mol Syst Biol. 2021 Sep 16;17(9):e10105. doi: 10.15252/msb.202010105 (PMC8444284; doi:10.15252/msb.202010105)
Supplement: Supplementary file 6 — Source Data for Figure 5 [file MSB-17-e10105-s004.zip › Figure5A_sourcedata/GSEA_3071/hallmarks_stateII.GseaPreranked.1623742536692/HALLMARK_MYC_TARGETS_V2.html]

Details for gene set HALLMARK\_MYC\_TARGETS\_V2[GSEA]

|  || Dataset | state23071 |
| Phenotype | NoPhenotypeAvailable |
| Upregulated in class | na\_pos |
| GeneSet | HALLMARK\_MYC\_TARGETS\_V2 |
| Enrichment Score (ES) | 0.5197383 |
| Normalized Enrichment Score (NES) | 2.5514941 |
| Nominal p-value | 0.0 |
| FDR q-value | 2.2727274E-4 |
| FWER p-Value | 0.001 |
Table: GSEA Results Summary

  

Fig 1: Enrichment plot: HALLMARK\_MYC\_TARGETS\_V2      
 Profile of the Running ES Score & Positions of GeneSet Members on the Rank Ordered List

  

| PROBE | GENE SYMBOL | GENE\_TITLE | RANK IN GENE LIST | RANK METRIC SCORE | RUNNING ES | CORE ENRICHMENT || 1 | MCM5 |  |  | 74 | 1.128 | 0.0364 | Yes |
| 2 | MCM4 |  |  | 81 | 1.110 | 0.0888 | Yes |
| 3 | CDK4 |  |  | 137 | 0.986 | 0.1230 | Yes |
| 4 | PLK4 |  |  | 205 | 0.870 | 0.1487 | Yes |
| 5 | MRTO4 |  |  | 228 | 0.847 | 0.1843 | Yes |
| 6 | TCOF1 |  |  | 234 | 0.838 | 0.2237 | Yes |
| 7 | NOP56 |  |  | 250 | 0.824 | 0.2599 | Yes |
| 8 | TMEM97 |  |  | 403 | 0.676 | 0.2553 | Yes |
| 9 | PA2G4 |  |  | 412 | 0.672 | 0.2859 | Yes |
| 10 | DCTPP1 |  |  | 477 | 0.638 | 0.3011 | Yes |
| 11 | SRM |  |  | 535 | 0.610 | 0.3166 | Yes |
| 12 | NPM1 |  |  | 544 | 0.605 | 0.3439 | Yes |
| 13 | NOLC1 |  |  | 546 | 0.603 | 0.3729 | Yes |
| 14 | HSPD1 |  |  | 606 | 0.576 | 0.3863 | Yes |
| 15 | NIP7 |  |  | 691 | 0.535 | 0.3915 | Yes |
| 16 | WDR43 |  |  | 742 | 0.518 | 0.4043 | Yes |
| 17 | UNG |  |  | 750 | 0.516 | 0.4276 | Yes |
| 18 | MYC |  |  | 790 | 0.502 | 0.4423 | Yes |
| 19 | PUS1 |  |  | 961 | 0.455 | 0.4225 | Yes |
| 20 | NOP2 |  |  | 983 | 0.448 | 0.4391 | Yes |
| 21 | IPO4 |  |  | 1006 | 0.443 | 0.4551 | Yes |
| 22 | PLK1 |  |  | 1009 | 0.442 | 0.4760 | Yes |
| 23 | IMP4 |  |  | 1129 | 0.412 | 0.4667 | Yes |
| 24 | UTP20 |  |  | 1356 | 0.366 | 0.4288 | Yes |
| 25 | LAS1L |  |  | 1366 | 0.364 | 0.4442 | Yes |
| 26 | MYBBP1A |  |  | 1371 | 0.362 | 0.4607 | Yes |
| 27 | PES1 |  |  | 1387 | 0.359 | 0.4744 | Yes |
| 28 | TBRG4 |  |  | 1415 | 0.353 | 0.4849 | Yes |
| 29 | PPRC1 |  |  | 1417 | 0.353 | 0.5018 | Yes |
| 30 | BYSL |  |  | 1473 | 0.344 | 0.5049 | Yes |
| 31 | EXOSC5 |  |  | 1513 | 0.339 | 0.5117 | Yes |
| 32 | PPAN |  |  | 1585 | 0.327 | 0.5101 | Yes |
| 33 | WDR74 |  |  | 1610 | 0.322 | 0.5197 | Yes |
| 34 | NOP16 |  |  | 1809 | 0.298 | 0.4854 | No |
| 35 | RRP12 |  |  | 1933 | 0.281 | 0.4687 | No |
| 36 | RRP9 |  |  | 2055 | 0.267 | 0.4518 | No |
| 37 | GRWD1 |  |  | 2066 | 0.265 | 0.4622 | No |
| 38 | SORD |  |  | 2077 | 0.265 | 0.4726 | No |
| 39 | NOC4L |  |  | 2124 | 0.260 | 0.4739 | No |
| 40 | SLC19A1 |  |  | 2206 | 0.252 | 0.4661 | No |
Table: GSEA details [plain text format]

  

Fig 2: HALLMARK\_MYC\_TARGETS\_V2: Random ES distribution      
 Gene set null distribution of ES for **HALLMARK\_MYC\_TARGETS\_V2**
